# Characterising a cohort of patients referred to a liaison psychiatry service from the Intensive Care Unit

**DOI:** 10.1192/bjo.2021.852

**Published:** 2021-06-18

**Authors:** Jennifer Hanks, Chun Chiang Sin Fai Lam, Isabel McMullen, Martin Parsons

**Affiliations:** South London and Maudsley NHS Foundation Trust

## Abstract

**Aims:**

This is descriptive study of a cohort of patients referred to a liaison psychiatry service from the intensive care department of a major London teaching hospital and trauma centre. The objective was to characterise key patterns in reasons for referral, nature of input, and gain a general sense of the workload. The rationale for collating this information was the consideration to developing a specific intensive care liaison service given the increasing evidence about the cognitive and mental health impacts of post-intensive care syndrome and the need for a coordinated management approach between stakeholders.

**Method:**

A cohort of 80 patients referred to liaison psychiatry service over a 6-month period from May to October 2020 was used. Descriptive statistics were used to characterise the patient's age, referring ward, reason for admission and referral, nature of input, number of reviews, previous engagement with mental health services, whether substance abuse or self-harm were related to the admission, and the destination upon discharge.

**Result:**

The age range of patients at point of referral was 25-80 years. For 25% of patients, this admission marked their first engagement with secondary mental health services and for around 50%, not only was a new diagnosis given during the admission, but there was no recorded history of any psychiatric diagnoses. Around 10% of patients were referred for management of delirium. Anxiety disorder accounted for the greatest proportion of diagnoses upon discharge, at 22%. There was much variability in the number of intensive care ward reviews carried out, ranging from one to over 10.

In 24%, self-harm led to presentation and 18% had comorbid substance misuse. Medication review was the single most common reason for referral in 13%, whereas requests for talking therapy and capacity assessments were 5% and 2% respectively. The vast majority of patients required a level of ongoing psychiatric input warranting community involvement or admission.

**Conclusion:**

This cohort often required detailed work-ups, new diagnoses and a high level of subsequent psychiatric management following discharge from hospital. The wide age range of patients meant that both working age and older adult liaison teams were involved in assessing referrals. Consideration could be given to a specific intensive-care liaison service due to the workload and complexity of needs, as well as the increasing awareness of the need for family support and early inclusion both for their benefit and that of the patient, particularly when the proportion of new diagnoses in this cohort is considered.

